# Development of a communication aid for explaining hypertrophic cardiomyopathy genetic test results

**DOI:** 10.1186/s40814-017-0205-0

**Published:** 2017-11-09

**Authors:** Yana Smagarinsky, Charlotte Burns, Catherine Spinks, Christopher Semsarian, Jodie Ingles

**Affiliations:** 1 Agnes Ginges Centre for Molecular Cardiology, Centenary Institute, Sydney, Australia; 2 Sydney Medical School, University of Sydney, Sydney, Australia; 3 Department of Cardiology, Royal Prince Alfred Hospital, Sydney, Australia

**Keywords:** Hypertrophic cardiomyopathy, Communication aid, Pathogenicity, Variant, Genetic counseling

## Abstract

**Background:**

Large gene panels are now commonplace for hypertrophic cardiomyopathy (HCM), increasing the yield of uncertain genetic findings. Few resources exist which aim to facilitate communication of HCM genetic test results. We sought to develop, pilot, and refine a communication aid for probands receiving HCM genetic test results.

**Methods:**

Development was a multi-step process involving expertise of a multidisciplinary team, literature review, and empirical experience. The aid went through an iterative revision process throughout the piloting phase to incorporate feedback. HCM probands attending a specialized multidisciplinary HCM clinic, aged ≥ 18 years and genetic test results available for disclosure between May and August 2016, or recently received their gene results (January–April 2015) were eligible. A purposive sampling strategy was employed, recruiting those attending clinic during the study period or those who could attend without difficulty.

**Results:**

We developed and pilot tested a genetic counsellor-led communication aid. Based on clinical expertise, the aid addresses (a) what genetic testing is, (b) implications for the patient, (c) reasoning for variant classification, and (d) implications for the family. Pilot data were sought to assess knowledge, feasibility, and acceptability using a self-report survey 2 weeks post-intervention. Twelve of 13 participants completed the follow-up questionnaire. Participants valued the individualised nature of the aid, recommended use of the aid, and indicated genetic knowledge, and family communication was better facilitated. Iterative modification of images helped to more simply depict important genetic concepts.

**Conclusions:**

We have developed a tool that is feasible, acceptable, and helpful to patients receiving genetic results. This is an important first step, and trial of the aid to assess effectiveness compared to usual care will follow.

## Background

Hypertrophic cardiomyopathy (HCM) is the most common genetic heart disease, affecting at least 1 in 500 people worldwide [1, 2]. Characterised by left ventricular hypertrophy in the absence of loading conditions such as hypertension, patients can be asymptomatic or suffer the most significant outcomes of heart failure and sudden cardiac death [3, 4]. HCM is inherited as an autosomal dominant trait, and genetic variants in at least 15 genes have been identified as causing HCM [5, 6]. Molecular diagnostic yield can vary based on clinical and family history characteristics [7–9], and many probands will undergo genetic testing and have no causative variant identified (i.e., an indeterminate result) [6]. The key utility of HCM genetic testing is for cascade genetic testing of asymptomatic relatives. Those found to be non-mutation carriers are excused from further clinical surveillance, alleviating unnecessary health costs and worry, thus making genetic testing a cost-effective component of HCM family management [10, 11].

Next-generation sequencing (NGS) technologies have allowed for the development of multi-gene DNA sequencing panel testing/cardiac gene chips. This testing has now become mainstream and allows for a large number of genes (often 50–100 genes) to be sequenced in a cost-effective and timely manner [6, 12]. Although NGS has greatly increased understanding of the genetic basis of HCM, the increased yield of rare variants has highlighted limitations of our current genetic knowledge and ability to effectively disseminate this information to patients [13–15]. The probabilistic nature of genetic test results means evidence for a variant is considered along a spectrum of pathogenic to benign status at a specific point in time. Ongoing reclassification based on new evidence is necessary and makes communication even more difficult [16, 17].

A diagnosis of HCM often has significant psychosocial challenges, with patients at greater risk of anxiety [18, 19], poor health-related quality of life [20–22], and in some sub-groups post-traumatic stress symptoms [19]. Communicating complex genetic information in this setting can therefore be problematic. Cardiac genetic counselors are skilled in delivering genetic information in a simple and sensitive way, though with increased complexity of the results, ways to better convey this information should be explored. Therefore, given the challenges in effectively communicating HCM genetic test results, we sought to develop, pilot, and refine a genetic counselor-led communication aid designed specifically for probands receiving a genetic test result for HCM. The objective was to determine feasibility and acceptability of the communication aid.

## Methods

### Overview of communication aid development and evaluation

**Fig. 1:**
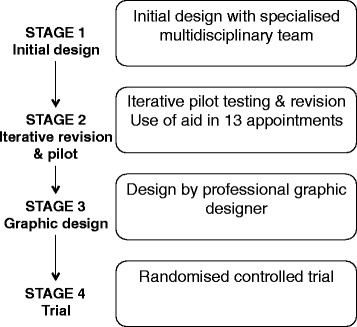
Study design––flowchart of the communication aid development and evaluation process

Development of the aid was a multi-step process. It was based on regular specialized multidisciplinary team meetings, informal literature review, and empirical experience (Fig. 1). This manuscript focuses on stage 1 (development) and stage 2 (piloting of the communication aid). Stage 3 involved input from a professional graphic designer. Finally, stage 4 is a genetic counselor-led randomized controlled trial to assess the effectiveness of the communication aid compared with usual care.

### Specialized multidisciplinary team

Usual practice for delivery of HCM genetic test results in the specialized multidisciplinary clinic setting involves a cardiologist and cardiac genetic counselor as previously described. Pre-test genetic counseling includes discussion regarding the potential outcomes of testing, implications for themselves, implications for their family members, and any psychosocial and insurance considerations. At time of result delivery, relevant points will be briefly re-iterated. Post-test counseling includes discussion of the findings, impact on their own medical management (which is usually minimal), and options now available for family members. This discussion occurs during the clinic appointment, which often includes follow-up cardiac investigations and consultation with the cardiologist. The process described above represents current care.

Development and iterative revisions of the aid involved a multidisciplinary team that included three experienced cardiac genetic counselors (JI, CB, CSp), a genetic counseling student (YS), and a cardiologist and Director of the HCM clinics (CS). The group had combined expertise in the clinical, psychosocial, and decision-making components of HCM genetic testing. They met frequently to discuss the initial development and later patient responses to the aid.

### Stage 1–development of the aid

The first draft of the aid was in the format of an A4 booklet. It focused on what the specialized multidisciplinary team considered to be the most important components of practice when disclosing HCM genetic test results. The team have extensive experience in providing HCM genetic testing to families for the last 15 years and have contributed numerous reviews about cardiac genetic counseling to the literature [15, 23, 24], including the key points of pre- and post-test cardiac genetic counseling (Table 1) which was used as a basis for the booklet [17]. Visual representation frameworks recommended by the International Patient Decision Aids Standards [25], patient information sheets from the Australian Genetic Heart Disease Registry (http://www.heartregistry.org.au), and other HCM-related literature helped guide the development of the images in the booklet and accompanying text [9, 26]. The development and pilot stage process was further assisted by consultation of literature reporting decision and communication aids relating to risk communication and genetic testing for breast and ovarian cancer [27, 28].

**Table 1:** Discussion points for pre-test and post-test cardiac genetic counseling [17]

Key issues	Discussion points
Genetic education	Clear explanation of genetic inheritance of the disease
Process of genetic testing	What was performed, how many genes were sequenced, limitations of our current technology
Explanation of all possible outcomes	Detailed discussion of potential outcomes of testing, i.e., pathogenicity of variants identified, potential for uncertainty requiring further investigation, potential for no variants to be identified
Clinical implications	Explain clinical implications of the gene result to the patient and their family members
Genetic implications	Explain the inheritance risks and genetic testing options for family members
Risk of reclassification	Families should be aware there is a small chance of reclassification of a variant, especially in cases where the evidence for causation is not as strong
Explore feelings and understanding	Ask how they feel about receiving their result, determine how family communication and dynamics will allow this information to be passed on. Gauge level of understanding of the information presented
Provide support with family communication and clinical or genetic screening	Offer assistance in conveying this information to family members, identifying local cardiologists to perform clinical screening and resources to explain the genetic testing options available to family members.

### Stage 2–piloting of the communication aid

An iterative piloting and revision process then followed, whereby the communication aid was used to facilitate the process of explaining genetic test results to HCM probands in genetic counselor-led appointments. Participants were those attending the HCM clinic for their regular appointments and due to receive their genetic test result. Patients with a diagnosis of HCM, who were adults and had sufficient English-skills (self-nominated) to complete the survey were eligible. The communication aid intervention was performed after the regular consult, during the time of result disclosure. Informed consent was obtained prior to the intervention, and for this phase of the study, participants only received the intervention (not usual care). The genetic counselor systematically described the images in the booklet and allowed for questions and comments.

At the completion of the piloting process, the revised communication aid was further developed by a professional graphic designer to produce the final version (stage 3).

#### Communicating genetic testing outcomes using visual formats

**Fig. 2:**
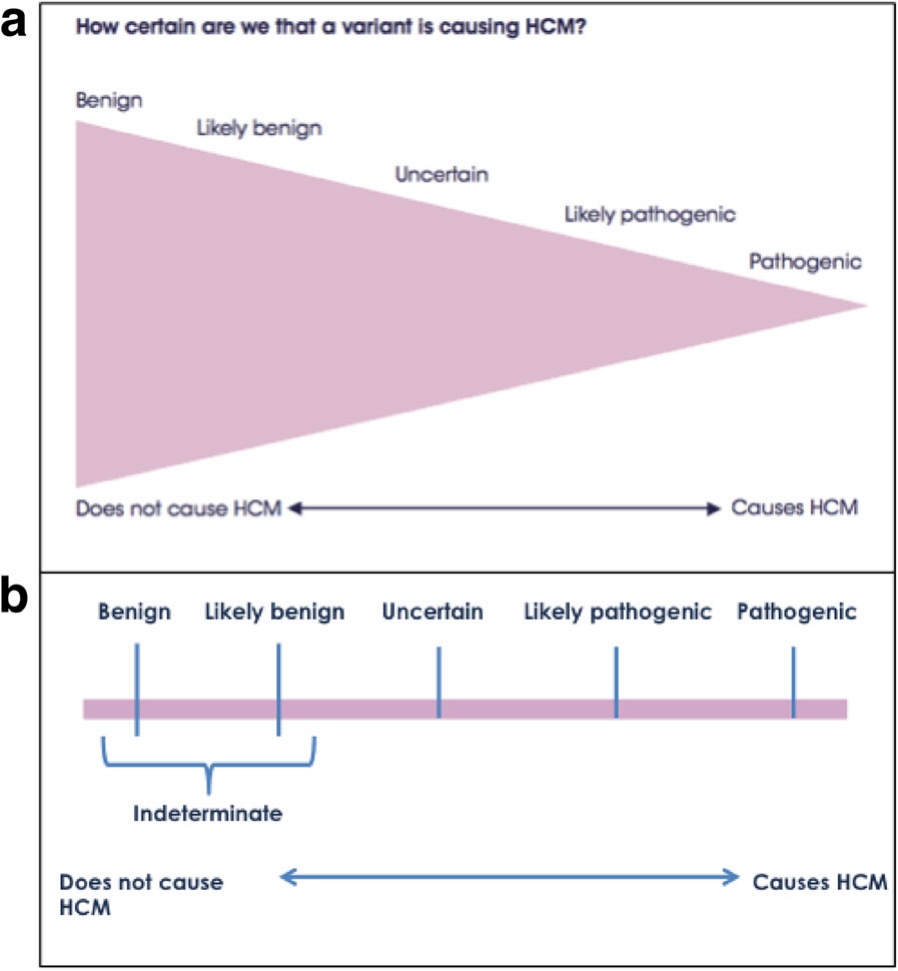
Probabilistic nature of genetic test results. **a** Genetic testing outcomes are initially depicted with respect to the frequency of which the types of variant classifications exist. Adapted from Maron BJ et al. (2012) [26]. **b** After several revisions during the piloting process, the figure was modified to help the proband more simply visualize variant classifications

Quantitative outcome information regarding the expected chance of finding a pathogenic or likely pathogenic variant was illustrated by an icon array [9, 25]. This was based on a figure previously reported and shows a wedge indicating clinical utility and the pathogenicity spectrum of variants (Fig. 2a) [26]. Members of the multidisciplinary team wrote and revised the text component of the booklet, and where possible, resources such as information sheets developed by the Australian Genetic Heart Disease Registry were used.

#### Take-home friendly for the patient and their family

The communication aid was developed to convey one key message per page opening. The bulk of the text was on the left pages of the booklet, while figures, tables, and images remained on the right. The genetic counselor could facilitate discussion easily by the use of the aids on the right, and at a later time when the booklet may be referred to, the text on the left would reinforce the information communicated during the session.

#### Tailoring to the individual

Based on multidisciplinary team discussions, an interactive element tailored to the specific genetic result of the patient was designed within the aid. Given the aim of the aid is to improve understanding of a given genetic result, the aid was designed to allow for the genetic counselor to write in the details of their genetic result (i.e., variants), its current classification, and the date the classification was made. In addition, the genetic counselor could tick boxes to indicate the key classification criteria pertaining to the variant, highlighting the level of evidence supporting pathogenicity. In cases where pathogenicity was uncertain and segregation studies are necessary, this aspect of the aid could help to reinforce the reasons for needing to engage other family members in the process. Segregation studies involve using additional family members to determine whether the variant tracks with disease through the family, it can be a very powerful piece of evidence for causation.

#### Plain language

Given the percentage of Australians with adequate health literacy rate is 43%, the content was designed assuming a low level of literacy [29]. The Flesch reading ease score of 59.1 and a Flesch-Kincaid grade level of 9.3 indicate the booklet is suitable for readers of a 9th grade level and above. Although design elements were based around encouraging the aid be re-read at home and passed on to family members, verbal delivery of the information with the use of the images by a genetic counselor was the primary goal, allowing potential to tailor delivery to the individual patient and their family members.

#### Information for at-risk relatives

The communication aid was developed to equip patients with enough knowledge and confidence to effectively communicate genetic risk information to their at-risk relatives. Therefore, the booklet was intended to be taken home, with sections highlighted in orange specifically for family members. Instructions for where family members can find specific information relevant to them were made clear throughout, enabling them to navigate the booklet easily.

A custom-designed table at the end of the booklet provided an opportunity for the genetic counselor to identify at-risk relatives by name and hand write recommended age-related clinical screening advice [30].

#### Communicating probabilistic genetic test results

The concept of a probabilistic test result, one that lies on a spectrum and can be subject to re-evaluation, was expected to be a new concept to most patients. Further, it may be necessary for families to act on this information, for example segregation studies to assess an uncertain variant or obtaining parental samples to determine de novo status. We created a figure to assist participants understand the concept more easily, with the image undergoing 2–3 subsequent revisions during the piloting process.

### Assessment of knowledge, feasibility, and acceptability

A self-report survey was sent to participant’s 2 weeks post-result delivery. The survey included a number of validated scales, targeted multiple-choice response, and open-ended questions. Satisfaction with services was assessed using the satisfaction with Genetic Counseling Scale [31]. Genetic knowledge was assessed using an amended version of the Breast Cancer Genetic Counseling Knowledge Questionnaire [32]. Participants were asked to evaluate the booklet on aspects such as length, amount of information, what they liked least and most, any suggestions for improvement, how many family members they had shown the booklet to, and if they would recommend genetic counselors use the booklet in appointments [28]. Satisfaction was measured with seven questions in regard to how helpful the booklet was in the context of HCM genetics on a 5-point Likert scale, adapted from Lobb et al. [27]. Open-ended questions concern how many immediate adult relatives the participant has informed about their genetic test result and reasons why they have or have not communicated the information to them. Participants were also asked to rate their ability to communicate this information to family members using a range “no difficulties” to “a lot of difficulties”. Responses to survey questions are shown descriptively.

### Data analysis

Descriptive statistics are presented, with categorical variables displayed as *n* (%) and continuous variables as mean ± standard deviation. Analyses were performed using GraphPad Prism 7.

## Results

### Study participants

Thirteen participants consented to the study and had their result delivered using the aid, with 12 (92%) surveys returned. One person declined participation. Table 2 outlines the clinical, sociodemographic, and variant characteristics of participants.

**Table 2:** Characteristics of the participants

Variable	N
Number of participants	12
Mean age, years (minimum–maximum)	46 (20–74)
Sex	
Male	9
Female	3
Education	
University degree	5
No university degree	7
Ethnicity	
White/Caucasian	12
Other	0
Genetic variant classification	
Indeterminate	5
Variant of uncertain significance	3^a^
Likely pathogenic	3
Pathogenic	1

^a^Two individuals had two uncertain variants (VUS) reported, and one had three VUS reported

### Satisfaction with genetic counseling

Patients were highly satisfied with the return of their results utilizing the communication aid as indicated by the scores from the satisfaction with genetic counseling scale. Instrumental 10.9 ± 2.6 and affective 11.3 ± 2.6 dimensions were assessed and indicated good levels of satisfaction (*N* = 12, score range 3–12). Single item scores (maximum score 4) relating to “expectations fulfilled” 3.8 ± 0.9, “satisfaction with information” 3.8 ± 0.9, and “overall satisfaction” 3.5 ± 0.9 all reflected high satisfaction with the genetic counseling service.

### Genetic knowledge

Table 3 shows the percentage of participants who gave correct answers to the genetic knowledge questions. Over 90% understood the concept of a 50/50 chance of passing on a pathogenic variant. Over 60% understood that an indeterminate result means family members are still at risk of disease, and over 80% understood that more evidence is needed to decide whether a variant of uncertain significance is causative or not.

**Table 3:** Genetic knowledge responses

Knowledge item	Answered correctly, *n* (%)
1. 50% of your genetic information is passed down from your mother (true)	8 (67)
2. 25% of your genetic information passed down from your father (false)	7 (58)
3. Each daughter and son has the same chance of developing HCM if one parent has HCM (true)	10 (83)
4. Genetic testing is the only way of finding out if someone has HCM (false)	10 (83)
5. An inherited HCM pathogenic variant is present in the DNA in every cell of the body (true)	5 (42)
6. There is more than one gene in humans, which if damaged, can cause HCM (true)	7 (58)
7. If a parent has an HCM pathogenic variant, each child has a 50% chance of inheriting it (true)	11 (92)
8. An individual who has a sibling with a HCM pathogenic variant has a 25% of also having the same variant (false)	5 (42)
9. If a person with HCM has an indeterminate genetic test result this means HCM is not inherited in their family (false)	8 (67)
10. A VUS may or may not be significant. More evidence and information is needed to know if this is important (true)	10 (83)

Abbreviations: *HCM* hypertrophic cardiomyopathy, *VUS* variant of uncertain significance

### Family communication

Ten out of 12 participants (83%) communicated their genetic test result to at least one adult family member (including spouse). Nine out of 12 (75%) participants rated their ability to communicate genetic information to family members with “no difficulties,” 3 out of 12 (25%) “some difficulties,” while no one reported “having a lot of difficulties.” Among the participants with indeterminate results, one participant had not disclosed her result to first-degree relatives. In the case of variants of uncertain significance, 2 out of 3 (67%) had not disclosed to first-degree relatives. Only the participant with an indeterminate result noted a reason for her non-disclosure, being that her relatives “are not interested”.

### Satisfaction, relevance, and emotional impact of the communication aid

All participants felt the booklet’s length and amount of information described was “about right.” No comments were made concerning any parts of the booklet needing further explanation or editing, with all but two participants stating they felt the booklet explained their situation “clearly” or “very clearly.” Only one participant (with a likely pathogenic result) stated the booklet made them feel “a little” worried or concerned. This same participant was the only one who did not read the booklet after taking it home as “it was explained to me at the clinic,” and further reported they had not disclosed the result to first-degree relatives. All participants recommended genetic counselors use the booklet when giving HCM test results back. Ten out of 12 (83%) participants showed the booklet to other family members. All satisfaction items scored ≥ 3.8 out of 5, indicating high satisfaction.

Overall participants had positive comments in regard to the booklet being used in a genetic-counselor-led appointment, with participants commenting that the information was “simplified for the common person.” Participants highlighted the value of the individualised nature of the aid––“clarity of information relating to me,” “[the] visual way [sic] in that when presented you can relate to the information better,” and “simple to read and very reassuring”.

### Key issues identified and corresponding revisions

The pilot test highlighted several important challenges in the communication of genetic test results, particularly relating to HCM genetics. Specifically, explaining the nature of the test results and how they may be re-evaluated in the future once more information is known, as well as the risk for the family members. We modified the communication aid to address these issues, as detailed below.

### Misinterpretation/misunderstanding of visualizing the nature of NGS test results

In the first draft of the communication aid, a triangular “wedge” image was used where the wider end represented the many benign variants that exist and the tip represented the rare pathogenic variants that exist (Fig. 2a).

The purpose of this figure was to help the patient visualize their genetic test result falling within this spectrum while also indicating the likelihood of certain types of variants being found (i.e., pathogenic variants most rarely). Initial piloting revealed patients did not easily understand this concept, and one participant thought it was a timeline of sorts, where more pathogenic variants are identified in time. This led us to edit the figure to portray a “sliding scale” (Fig. 2b) with the test results placed along it, thus facilitating discussion of reclassification, particularly for the participants who had a VUS result. Removing the additional information represented by the wedge allowed a simpler message and clearer understanding.

### Participants with indeterminate results felt “left out”

As an indeterminate result technically is not visually represented within the spectrum of results (as no suspicious variant was identified in the first instance), this led to participants questioning where they belonged, i.e. where they “fall” on the spectrum of genetic test results (Fig. 2).

**Fig. 3:**
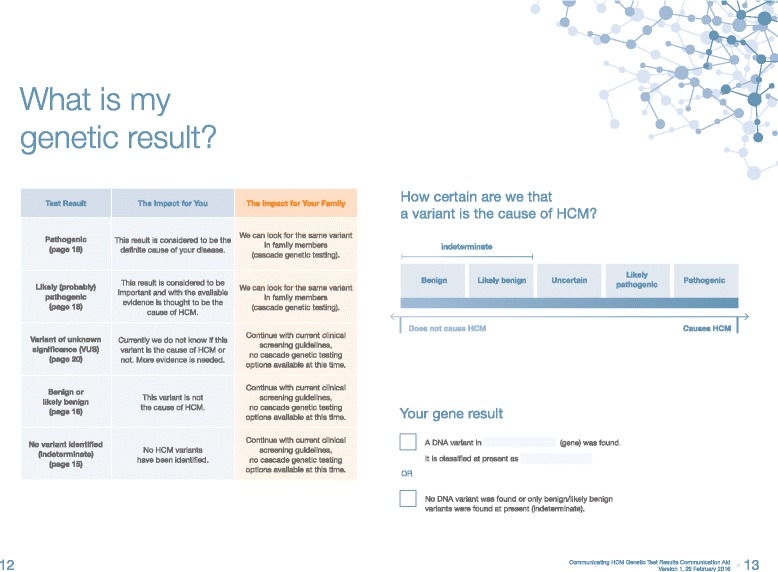
Example of an opening of the final version of the booklet. The text is purposely placed on the left, with visuals and interactive images on the right to facilitate discussion with the genetic counselor. The text can be referred to a later stage and reinforces key points

In the specialized HCM Clinic, variants found to having a frequency > 1% are not reported, which is stated on the report. To make the booklet more inclusive for participants with indeterminate results, we added an overarching bracket to the benign and likely benign end of the “sliding scale” to highlight that benign and likely benign variants were possibly found but were not reported. We also added a further statement that can be “ticked” stating “no DNA variant was found, or only benign/likely benign variants found at present (indeterminate)” (Fig. 3).

### Chronological versus non-chronological ordering of the booklet

We aimed to create a communication aid that could be read page-to-page in chronological order, as well as offering the reader the opportunity to go directly to a desired page or section. One participant noted that the chapter titles were confusing, so the chapter titles were edited to better allow the reader to understand which section they had turned to, without needing to refer back to the contents page.

## Discussion

This paper describes the development and evaluation of a communication aid, designed to facilitate effective discussion of HCM genetic test results with patients. This manuscript focuses exclusively on the development and pilot stages of the study, where iterative revision of the booklet content for feasibility and usability were performed to optimize patient understanding. We demonstrated the communication aid was well received by participants in facilitating a genetic test result discussion, with high satisfaction levels measured overall. Participants reported little difficulty in explaining the result to their family, with most showing the booklet to other family members. Genetic knowledge was good, and overall, the feedback indicates communication aids in the setting of HCM can play an important role in improving patient communication and understanding.

While most participants commented favorably regarding the personalized nature of the booklet, those with indeterminate results required some modifications to make the booklet more inclusive, and we were able to address this during the iterative revisions. The participant with an indeterminate result who chose not to disclose to family members commented in the questionnaire that the information was not useful (this being the first draft of the booklet)––“I don’t know what to do with the information given to me…Perhaps there was a curse put on me.” This response is perhaps reflective of overarching issues surrounding the communication of an uncertain result, i.e., indeterminate or variant of uncertain significance, highlighting the ways in which uncertainty is perceived and the unfortunate lack of actionable qualities [33]. Uncertainty in genetics has been explored in a range of settings, and the literature highlights that patients have difficulty comprehending the meaning of an uncertain result, how to proceed with the information and whether or not to inform family members of the result [34, 35]. This is particularly evident in studies demonstrating fewer patients choosing to disclose such a result with family members compared to those with a pathogenic result [36, 37]. In spite of this, understanding that HCM is a heritable condition with a 50% risk to first-degree relatives should be communicated with or without a conclusive genetic result.

Unlike other medical settings, an HCM gene result will not often impact on the patient’s own clinical management or therapy options. For example, an indeterminate or VUS result is unlikely to change prognosis and available treatment options. Regardless, facilitating an informed discussion of indeterminate or VUS results is imperative. Understanding that the lack of a gene result still requires family members to undergo routine clinical screening, or that a VUS does not mean they have some type of “atypical” disease that may prompt unnecessary worry is important. Such a discussion also gives an opportunity to address issues surrounding feelings of uncertainty, which may affect the well-being of the patient and present as barriers to family communication [36].

Research regarding the use of decision aids by health professionals has shown they help increase knowledge levels, stimulating patients to feel more informed and have clearer values [38]. In this study, over half of the participants answered all but one of the questions relating to NGS testing outcomes correctly. Whether the booklet improves knowledge will be answered by the randomised controlled trial, which has commenced (Australia and New Zealand Clinical Trial Registration; ACTRN12617000706370). As genetic counselors empower people to make informed decisions by educating and instilling knowledge regarding genetic contributions to disease, this booklet shows great promise to facilitate genetic counselors in providing optimal care [39].

Considering the key role of HCM genetic test results in family management, we were motivated to ensure the booklet information was easily accessible to at-risk relatives who were not present at the results disclosure appointment. We encouraged probands to pass the booklet on to family members allowing them to read and discuss the implications of their result. In order to communicate genetic results or risk information, the proband must have adequate understanding of the information received. There is limited literature that explores the use of communication aids in the public health sector, let alone in the genetic sector. The majority of studies undertaken explore the development and use of communication and decision aids specifically tailored to cancer risk, screening methods, and genetic testing for people in oncology consultations [27, 28, 40, 41].

## Conclusions

HCM genetic testing in the era of NGS technology is increasingly complex. Genetic counselors and specialized multidisciplinary teams communicating results require effective tools to increase knowledge and understanding and equip probands with adequate skills to communicate this to family members. We have developed and piloted a resource that addresses these issues and have shown use of the aid is acceptable to patients within the specialized multidisciplinary clinic. Participants valued the individualized nature of the aid, recommended use of the aid, and indicated genetic knowledge, and family communication was facilitated by the communication aid. A randomized controlled trial using this piloted booklet will determine whether this is an effective tool in better communicating complex genetic information to patients and their families with HCM.

## Abbreviations

HCM: Hypertrophic cardiomyopathy
NGS: Next-generation sequencing
VUS: Variant of uncertain significance
